# Expression of VjbR under Nutrient Limitation Conditions Is Regulated at the Post-Transcriptional Level by Specific Acidic pH Values and Urocanic Acid

**DOI:** 10.1371/journal.pone.0035394

**Published:** 2012-04-17

**Authors:** Gastón M. Arocena, Angeles Zorreguieta, Rodrigo Sieira

**Affiliations:** 1 Fundación Instituto Leloir - IBBA-CONICET, Buenos Aires, Argentina; 2 Departamento de Química Biológica, Facultad de Ciencias Exactas y Naturales, Universidad de Buenos Aires, Buenos Aires, Argentina; Universidad Nacional, Costa Rica

## Abstract

VjbR is a LuxR homolog that regulates transcription of many genes including important virulence determinants of the facultative intracellular pathogen *Brucella abortus*. This transcription factor belongs to a family of regulators that participate in a cell-cell communication process called quorum sensing, which enables bacteria to respond to changes in cell population density by monitoring concentration of self produced autoinducer molecules. Unlike almost all other LuxR-type proteins, VjbR binds to DNA and activates transcription in the absence of any autoinducer signal. To investigate the mechanisms by which *Brucella* induces VjbR-mediated transcriptional activation, and to determine how inappropriate spatio-temporal expression of the VjbR target genes is prevented, we focused on the study of expression of *vjbR* itself. By assaying different parameters related to the intracellular lifestyle of *Brucella*, we identified a restricted set of conditions that triggers VjbR protein expression. Such conditions required the convergence of two signals of different nature: a specific pH value of 5.5 and the presence of urocanic acid, a metabolite involved in the connection between virulence and metabolism of *Brucella*. In addition, we also observed an urocanic acid, pH-dependent expression of RibH2 and VirB7, two additional intracellular survival-related proteins of *Brucella*. Analysis of promoter activities and determination of mRNA levels demonstrated that the urocanic acid-dependent mechanisms that induced expression of VjbR, RibH2, and VirB7 act at the post-transcriptional level. Taken together, our findings support a model whereby *Brucella* induces VjbR-mediated transcription by modulating expression of VjbR in response to specific signals related to the changing environment encountered within the host.

## Introduction

*Brucella* is a genus of Gram-negative facultative intracellular bacteria that comprises several species. They are able to invade and replicate within various cell types of their mammal hosts, including macrophages. Bacteria belonging to this genus are the causative agent of brucellosis, a zoonotic disease that affects reproduction of infected animals due to colonization of testicular, placental and fetal tissues [1]. In humans, the acute phase of brucellosis produces debilitating symptoms that include undulant fever, whereas the chronic phase is characterized by serious clinical manifestations such as endocarditis and neurological disorders [1]. *Brucella abortus*, *Brucella suis*, and *Brucella melitensis* are the species that have more impact on public health and animal industry since, in addition to humans, they infect cattle, swine, and goat, respectively.

*Brucella* circumvents the bactericidal mechanisms of the host cells by modifying the kinetics of acquisition of organelle-specific marker proteins of the so called *Brucella*-containing vacuole (BCV). In this way, these bacteria actively control the BCV intracellular trafficking, avoid degradation by lysosomes, and promote the formation of a replication-permissive compartment in an endoplasmic reticulum (ER)-derived organelle, where they multiply [2]. Shortly after *Brucella* enters the host macrophage, the BCV undergoes limited and controlled interactions with acidic LAMP-positive compartments, which lowers luminal pH to values near 4.5 [3], [4]. Early studies demonstrated that this acidification process is essential for the intracellular survival, since neutralization of the vacuolar pH was detrimental for bacterial replication within macrophages [3]. Accordingly, it was subsequently found that acidification of the BCVs is necessary for the intracellular expression of important virulence-associated genes such as the *virB* operon [5], [6]. The *virB* genes code for a Type-IV Secretion System (T4SS) that mediates early interactions between BCVs and ER-derived membranes, which are required for the control of the intracellular trafficking of *Brucella* [7]. In addition, the T4SS VirB was recently found to be involved in translocation of specific substrates into the host cell cytoplasm [8], [9], [10]. Transcription of the *virB* genes is induced immediately after internalization of *Brucella* into the host cell, reaching a maximum level of expression at 5 hours post infection [5], [11]. Subsequently, it is rapidly repressed prior to the onset of bacterial replication [4], [11]. HutC, a well-known transcriptional regulator of the histidine utilization (Hut) pathway, participates in *virB* regulation as a coactivator by interacting with a specific binding-site in the *virB* promoter [12]. Besides regulating intracellular *virB* expression, HutC is also necessary for transcription of the *virB* genes in bacteria cultured *in vitro* at pH 4.5 in the presence of urocanic acid, the inducer of histidine catabolism [12]. In addition to HutC, several transcriptional regulators were shown to be involved in regulation of the *virB* genes [13]. Among them, the LuxR-homolog VjbR plays a preponderant role, since deletion of *vjbR* completely abrogates *virB* expression and leads to an avirulent phenotype [14].

VjbR belongs to a family of transcriptional regulators that participate in a cell to cell communication process called quorum sensing (QS), which enables bacteria to respond to changes in cell population density by monitoring the concentration of self produced autoinducer signals. Both the amino acid sequence of the DNA-binding domain and the architecture of its target DNA-sequence revealed that VjbR is evolutionarily related to BisR and MrtR, two LuxR-type transcription factors involved in regulation of symbiotic plasmid conjugation and nodulation of *Rhizobium* and *Mesorhizobium* species [15], [16], [17]. Interestingly, VjbR exhibits several unusual features that, taken together, distinguish it from the long list of known LuxR-family members. First, VjbR is one of the few QS-related regulators that bind to DNA and regulate transcription in the absence of any autoinducer signal [14], [15], [18]. Second, it belongs to the group of orphan LuxR regulators [19], since *Brucella* lacks genes for synthases of acyl-homoserine lactone (AHL) QS-signaling molecules. However, although the mechanism of synthesis has not been elucidated so far, an *N*-dodecanoyl-homoserine lactone (C_12_-AHL) molecule has been identified at low amounts in culture supernatants of *B. melitensis* [20]. Furthermore, VjbR retains a functional AHL-responsive domain, since the exogenous addition of C_12_-AHL dissociates the regulator from DNA, thus abrogating expression of the *virB* operon and the VjbR-mediated regulation of many additional genes [14], [15], [21]. These evidences support a model wherein a signaling-molecule could inhibit the VjbR DNA-binding activity *in vivo*, which may account for the rapid kinetics of the switch off observed in the intracellular expression of the *virB* genes [4], [11]. However, nothing is known about the mechanisms involved in the rapid VjbR-mediated induction of *virB* expression following internalization, or how an inappropriate spatio-temporal expression of the VjbR target genes is prevented. Based on the hypothesis that an induction of VjbR per se would lead to transcriptional activation of the downstream target genes, we aimed to investigate the regulation of the *vjbR* gene itself. Analyses of protein expression, promoter activities and determinations of mRNA levels performed under conditions that resemble those encountered by the bacterium within the host cells allowed us to identify signals that, when they converge, promote expression of VjbR and other virulence determinants of *Brucella*. Our results demonstrate that the signal-mediated induction of VjbR expression occurs at the post-transcriptional level, which supports a model whereby *Brucella* is able to control VjbR-mediated transcription by modulating the levels of VjbR in response to specific signals related to the changing environment encountered within the host cell.

## Materials and Methods

### Ethics Statement

This study was carried out in strict accordance with the recommendations in the Guide for the Care and Use of Laboratory Animals (National Academy Press, Washington DC: 2010) and those from the Principles for Biomedical Research involving animals developed by the Council for International Organizations of Medical Sciences. The protocol AZ16/09 was approved by the Institutional Animal Care and Use Committee (IACUC) of the Fundación Instituto Leloir (OLAW assurance number A5168-01). All efforts to minimize suffering were made.

### Strains, media, and growth conditions

Wild type *B. abortus* 2308 or the isogenic deletion mutant strain *B. abortus ΔvjbR* were grown in triptic soy broth (TSB), or in minimal medium MM1 [12] at pH 4.5, 5.5, or 7.0. *cis*-urocanic acid (Sigma) or L-glutamic acid (Sigma) were added to a final concentration of 5 mM, and media were sterilized by filtration through a 0.22 µm-pore diameter filter unit (Millipore). Media were supplemented with kanamycin (50 µg.ml^−1^) as needed. Growth curves for protein analyses and β-galactosidase activity determinations were carried out as follows: *B. abortus* strains grown in TSB-agar plates for 3–4 days were suspended in 25 ml of TSB to an OD_600_ of 0.05 into a 125 ml-polycarbonate Erlenmeyer flask, and were cultured at 37°C in a rotary shaker (250 rpm). Under these conditions, bacterial strains used in this work grew exponentially with a duplication time of about 3.5 hours, and reached a maximal OD_600_ of 3.7 at the stationary phase.

### Construction of the *B. abortus ΔvjbR* deletion mutant strain

Two PCRs were carried out using *Pfx* (Invitrogen), genomic DNA of *B. abortus* 2308 as template, and primers vjbRa (5′-GGACTAGTGTCGCCTTGCGTTACAAGC-3′) and vjbRCOMa (5′-TCGATACTCTCCTTCTTGCAGCTCGTCTGATCAACATGGTC-3′), or vjbRCOMb (5′-CTGCAAGAAGGAGAGTATCGACATTGGAAATATCCTTGGTGA-3′) and vjbRb (5′-GGACTAGTCGCCAGCATGAACGACATC-3′). Both PCR products, corresponding to flanking regions of *vjbR*, were annealed and used as templates for a PCR performed with primers vjbRa and vjbRb. The product was digested with SpeI and cloned into plasmid pK18mob-*sacB* [22], generating plasmid pK18mob-*sacB-ΔvjbR*, which was transferred to *B. abortus* 2308 by biparental conjugation. Kanamycin^r^ colonies were selected as single-homologous recombinants. Selection with sucrose, excision of plasmids and generation of deletion mutants was performed as described previously [11]. PCR analyses of kanamycin^s^ colonies were carried out with primers vjbRa and vjbRb to identify clones that contain the deletion of *vjbR*. The absence of VjbR in total protein extracts from strain *B. abortus ΔvjbR* was verified by western blot analyses carried out with anti-VjbR antibodies.

### Western blot analyses

Bacteria were grown in rich medium TSB until OD_600_≤1.5. Subsequently, cultures were centrifuged and suspended in different media, as indicated. After 4 h of incubation, OD_600_ was determined and samples equivalent to 3 ml of OD_600_ = 1.0 were centrifuged, suspended in 180 µl of sodium dodecyl sulfate (SDS) loading gel buffer and incubated at 100°C for 5 min. 12 µl of each bacterial cell lysate was subjected to electrophoresis in a 10% -polyacrylamide gel for detection of VjbR, RibH2, HutC or RibH1; or in a 12.5% SDS-polyacrylamide gel for detection of VirB7. Proteins were transferred to PVDF membranes (Amersham), and loading equivalency was corroborated by Ponceau S staining immediately after each protein transfer. Proteins were detected using a mouse anti-VjbR (dilution 1∶1,000) polyclonal antibodies (which were generated in this work by immunization of C57 Black mice with recombinant VjbR protein) [15], or mouse monoclonal anti-RibH2 (dilution 1∶1,000) [23], rabbit polyclonal anti-VirB7 (dilution 1∶1,000) [11], mouse polyclonal anti-HutC (dilution 1∶1,000) [12], or rabbit polyclonal anti-RibH1 antibodies (dilution 1∶600) [23]. Subsequently, peroxidase-conjugated secondary antibodies anti-mouse (dilution 1∶30,000) (Santa Cruz) or anti-rabbit immunoglobulins (dilution 1∶10,000) (Sigma) were used to develop membranes with ECL Plus Western Blotting Detection System (Amersham). Chemiluminiscence was detected by autoradiography.

### Construction of *lacZ* transcriptional fusions

The *lacZ* gene was amplified by PCR using primers lacZupSphI (5′-GGGCATGCATCGATAGATCTCGAGATCC-3′) and lacZdownSphI (5′-GGGCATGCTTATTTTTGACACCAGACCAAC-3′), and plasmid pAB2001 [24] as template. The PCR product was cloned into pGEM-T Easy (Promega). The resulting plasmid (pGEM-*lacZ*) was digested with SphI, releasing a 3.07-kbp fragment which was cloned into plasmid pK18mob [22] in the SphI site and oriented towards the HindIII restriction site of the polylinker, generating plasmid pK18mob-*lacZ*.

A 277-bp region corresponding to sequences located 1 nucleotide upstream of the *vjbR* coding region was amplified by PCR using primers vjbRupBamHI (5′-CGGGATCCGTAGAGAATGTGTTCCAAAG -3′) and vjbRdownBamHI (5′-CGGGATCCTGGAAATATCCTTGGTGATG -3′), and *B. abortus* 2308 genomic DNA as template. The PCR product was cloned into pGEM-T Easy (Promega). The resulting plasmid (pGEM- P*_vjbR_*) was digested with BamHI, and the released fragment was cloned into plasmid pK18mob-*lacZ* in the BamHI site and oriented towards the HindIII restriction site of the polylinker, generating plasmid pK18mob-P*_vjbR_*-*lacZ*.

Genomic sequences of about 280 bp corresponding to the promoter regions of *ribH1* or *ribH2* were amplified using primers PribH1up (5′-GTTTCCCGATGTACGGCTAT-3′) and PribH1down (5′-ACTGGCAATAGGGACAAC-3′), or PribH2up (5′-ACCTAGGATAGGCTCAAACC-3′) and PribH2down (5′- CGCGCACCATTGCGCGAG-3′), respectively; and *B. abortus* 2308 genomic DNA as template. The PCR products were cloned into pGEM-T Easy (Promega). The resulting plasmids (pGEM- P*_ribH1_* or pGEM- P*_ribH2_*) were digested with EcoRI, and the released fragments were cloned into plasmid pK18mob-*lacZ* in the EcoRI site, and oriented towards the HindIII restriction site of the polylinker, generating plasmids pK18mob-P*_ribH1_*-*lacZ* or pK18mob-P*_ribH2_*-*lacZ*.

Strains *B. abortus* P*_virB_*-*lacZ* and *B. abortus* P*_hut_*-*lacZ* were obtained from a previous study [12]. Strains *B. abortus* P*_vjbR_*-*lacZ* and *B. abortus ΔvjbR* P*_vjbR_*-*lacZ* were generated by introducing plasmid pK18mob-P*_vjbR_*-*lacZ* into strains *B. abortus* 2308 or *B. abortus ΔvjbR*, respectively, by biparental conjugation. Chromosomal single recombinants were selected using kanamycin. Strains *B. abortus* P*_ribH1_*-*lacZ* and *B. abortus* P*_ribH2_*-*lacZ* were generated by introducing plasmids pK18mob-P*_ribH1_*-*lacZ* or pK18mob-P*_ribH2_*-*lacZ*, respectively, into strain *B. abortus* 2308 by biparental conjugation, and selecting chromosomal single recombinants with kanamycin. The proper insertion of the *lacZ*-transcriptional fusions into the chromosomes of *Brucella* was corroborated by colony PCR using primers corresponding to the upstream end of each construct used and primer lacZ6143 [12]

### β-galactosidase activity determinations

Bacteria were grown in rich medium TSB until OD_600_≤1.5. Subsequently, cultures were centrifuged and suspended in different media, as indicated. After incubations of 4 h, β-galactosidase activities were determined with whole cells as described [25], with the following modifications: after incubation for 10 min with o-nitrophenyl-β-D-galactopyranoside, reaction mixtures were centrifuged before determinations of *A* ^420^. β-galactosidase activity was expressed as [*A* ^420^/volume×OD_600_]×100.

### Quantitative real-time PCR analysis of gene expression

Strain *B. abortus* 2308 was grown in rich medium TSB until OD_600_≤1.5. Subsequently, cultures were centrifuged and suspended for 4 h in MM1 at pH 5.5, with or without 5 mM urocanic acid. Subsequently, bacteria were harvested and total RNA was isolated using the Illustra RNAspin Mini RNA Isolation Kit (General Electric). Samples corresponding to 1 µg of RNA were treated with RQ1 RNase-free DNAse (Promega) and reverse transcribed with SuperScript III (Invitrogen) and 0.5 µg random hexamers (Invitrogen) per µg of RNA. A negative control reaction was also prepared by omitting the addition of reverse transcriptase. Transcript levels were measured in a Mx3005P quantitative-PCR system (Stratagene) using cDNAs as template, SYBR Green PCR Master Mix (Applied Biosystems) and primers qPCRvjbRup (5′-GGTTTTTCAGGAAGACGCTC-3′) and qPCRvjbRdown (5′-AAGATTTCCCAGGCCGTGC-3′); qPCRhutCup (5′-TTTGAACACGAGCTGACCGA-3′) and qPCRhutCdown (5′-TGCGATTGCGGGAACGACA-3′); qPCRvirB7up(5′-CCTTGCTTTTGTCGCCACG-3′) and qPCRvirB7down (5′-GTAAGTGTCAACGGGGTTAG-3′); qPCRribH2up (5′-GCGGCCTTCGTGATCGAC-3′) and qPCRribH2down (5′- TGCTTTCATGGAAATGGTGC-3′); qPCRribH1up (5′- CGGCAACGATTTCCTTCGC-3′) and qPCRribH1down (5′- GGCAGGATTCATTGGACAC-3′); or qPCR-IF-1up (5′- ACTAGAACCTTGTCACCGGC-3′) and qPCR-IF-1down (5′- ATGGCGAAAGAAGAAGTCCT -3′); which amplify sequences of about 135 pb located at the coding regions of *vjbR*, *hutC*, *virB7*, *ribH2*, *ribH1*, and *IF-1*, respectively. After amplification, each reaction was analyzed by performing a dissociation curve to ensure the absence of primer dimers or unspecific amplifications, which revealed that all fluorescent signals obtained corresponded to single amplicon species whose melting temperatures ranged from 83.3 to 87°C, depending on the primers used. Transcript levels were normalized to that of the *IF-1* houskeeping gene, and were quantified using the relative quantity dRn method of the Mx3005P software, displaying values relative to samples treated with 5 mM urocanic acid.

### Statistical analysis

A two-tailed Student's *t* test was used to assess statistical differences between two experimental data sets for all quantitative results reported in this study. A value of *P*<0.05 was considered to indicate statistical significance.

## Results

### Analysis of expression of VjbR under standard culture conditions

**Figure 1:**
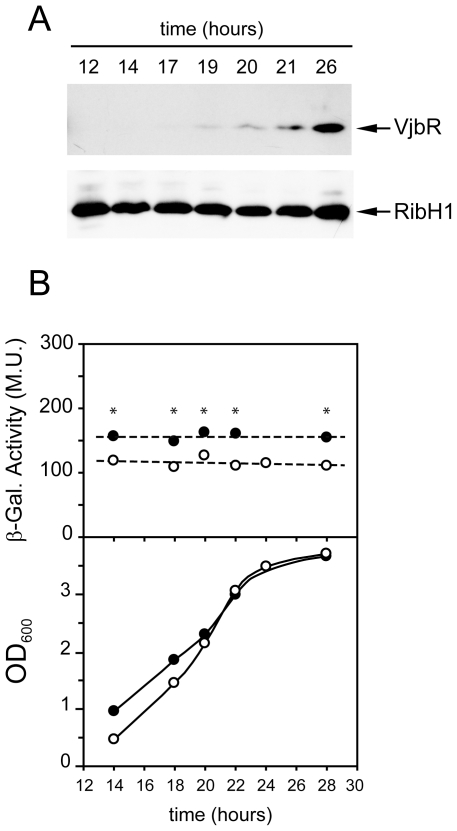
Analysis of expression of VjbR under standard culture conditions. (A) Western blot analysis. *B. abortus* 2308 wild type strain was grown in rich medium (TSB). At different times, optical density at 600 nm (OD_600_) was determined, and samples were subjected to 10% SDS-PAGE, transferred to PVDF membranes, and developed with anti-VjbR or anti-RibH1 polyclonal antibodies. The corresponding optical density at 600 nm (OD_600_) of each sample was 0.5, 0.7, 1.7, 2.6, 2.95, 3.5, and 3.6, respectively. Results from a representative of two independent experiments are shown. (B) Analysis of the *vjbR* promoter activity. Strains *B. abortus* P*_vjbR_*-*lacZ* (white circles) or *B. abortus ΔvjbR* P*_vjbR_*-*lacZ* (black circles) were cultured in TSB. OD_600_ and β-galactosidase activities were determined along the growth curve at the indicated times. Values are means ± standard deviations of duplicate samples from a representative of two independent experiments. Dotted lines indicate the best linear fit to the data. *, *P*<0.05.

Initially, we analyzed VjbR protein expression in cultures of the wild type strain *B. abortus* 2308 grown in rich medium. To this end, equal amounts of bacteria were harvested at different stages of the growth curve in tryptic soy broth (TSB), and the extracts were analyzed by western blot using antibodies against VjbR. As a control for protein loading, the same extracts were also analyzed using antibodies against RibH1, a lumazine sinthase (LS) isoenzyme that exhibits constitutive expression in *B. abortus* (F. Goldbaum, personal communication). As shown in Fig. 1A, VjbR was induced in the stationary phase with a temporal pattern of expression similar to that observed previously by other groups [26], whereas the levels of RibH1 remained constant throughout the entire growth curve. To determine whether the increase of VjbR protein expression was a result of transcriptional activation, we constructed a strain that carries a single-copy chromosomal transcriptional fusion construct consisting of a *lacZ* reporter gene inserted 1 nucleotide upstream of the *vjbR*-coding sequence. The resulting strain, *B. abortus* P*_vjbR_*-*lacZ*, was grown in TSB and β-galactosidase activities were determined along the growth curve. As shown in Fig. 1B, the sequences that direct *vjbR* expression showed a constant promoter activity throughout the experiment, which indicated that transcription of *vjbR* is constitutive under these standard culture conditions, and suggested that the observed stationary phase-dependent induction of VjbR protein expression was due to a post-transcriptional regulatory mechanism. The analysis of the *vjbR* promoter performed in a *B. abortus ΔvjbR* mutant strain also showed a constitutive β-galactosidase activity, which remained 1.3-fold higher than that of the wild type background in all phases of growth (Fig. 1B). These observations differ from those reported in other studies performed in *B. melitensis* [27], [28], which suggests that regulation of the *vjbR* promoter probably differs among *Brucella* species.

### Analysis of expression of VjbR under conditions related to the intracellular life of *Brucella*

**Figure 2:**
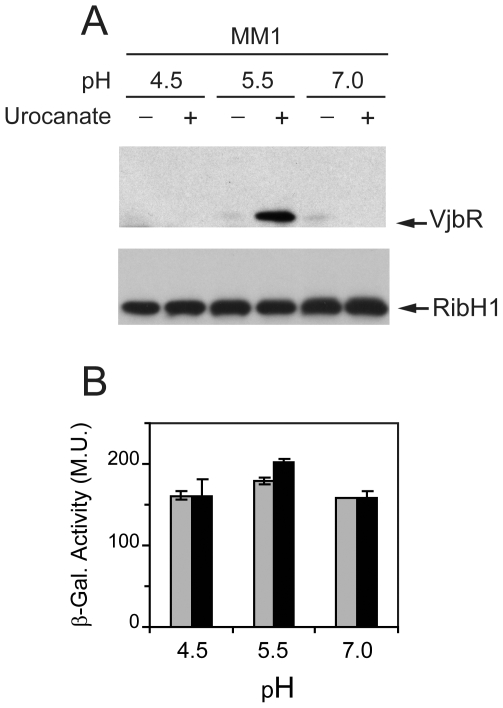
Analysis of expression of VjbR under different acidic and metabolic conditions. (A) *B. abortus* 2308 wild type strain was grown in TSB until OD_600_≤1.5. Subsequently, cultures were centrifuged and bacteria were resuspended in medium MM1 at pH 4.5, 5.5, or 7.0; in the absence or presence of 5 mM urocanic acid, as indicated. After incubation under different conditions, OD_600_ was determined, and samples were analyzed by western blot using anti-VjbR or anti-RibH1 polyclonal antibodies. Results are representative from one of three independent experiments. (B) Analysis of the *vjbR* promoter activity. Strain *B. abortus* P*_vjbR_*-*lacZ* was cultured in TSB and shifted to MM1 at pH 4.5, 5.5, or 7.0; in the absence (gray bars) or presence (black bars) of 5 mM urocanic acid. Subsequently, β-galactosidase activities were determined. Values are means ± standard deviations of duplicate samples from a representative of three independent experiments.

We next asked whether the intracellular conditions that *Brucella* encounters within the host cell might trigger expression of VjbR. To answer this question, we performed experiments using bacteria grown in TSB to an OD_600_ lower than that at which VjbR expression first appears (see materials and methods). Subsequently, bacteria were shifted to different media prepared according to the current knowledge of the variables that characterize the BCVs [3], [5], [29], which reproduce a nutrient-poor environment at different pH values to which *Brucella* could be exposed during the acidification of the vacuole. Taking into account that histidine catabolism is linked to induction of the intracellular survival-related *virB* genes [12], we also assayed the levels of VjbR in the absence or presence of urocanic acid, the inducer of the Hut pathway. After incubation of bacteria under these different media for 4 hours, the effect of each treatment on protein expression was evaluated by western blot. As shown in Fig. 2A, VjbR protein was induced only under a restricted set of conditions, indicating that expression of this LuxR homolog is tightly regulated and depends on the convergence of two signals that comprise pH 5.5 and the presence of urocanic acid. It was also observed that the levels of RibH1 remained constant in all extracts, indicating that the effect of urocanic acid and pH 5.5 was specific for VjbR expression (Fig. 2A). Determination of CFU ml^−1^ showed no differences in bacterial viability between treatments at pH 5.5 or 7.0 (Figure S1). In contrast, there was a gradual drop in viability at pH 4.5 in the absence of the carbon source, whereas the addition of urocanic acid somehow exerted a protective effect and maintained viability at levels similar to those obtained at higher pH values (Fig. S1). Therefore, these observations indicated that induction of VjbR at pH 5.5 in the presence of urocanic acid was a result of regulation of gene expression rather than effects caused by differences in viability.

**Figure 3:**
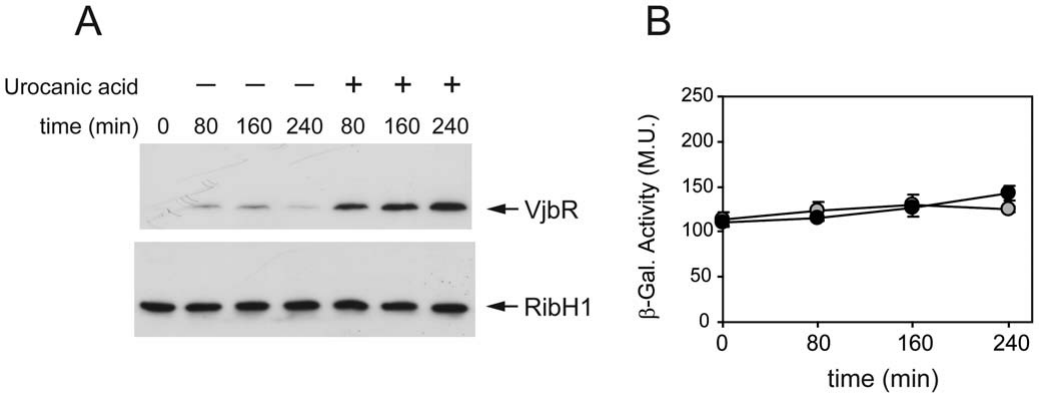
Analysis of expression of VjbR at pH 5.5 between 0 and 4 hours. (A) Wild-type strain *B. abortus* 2308 was grown in TSB until OD_600_≤1.5. Subsequently, cultures were centrifuged and bacteria were resuspended in medium MM1 at pH 5.5 in the absence or presence of 5 mM urocanic acid. At the indicated times, OD_600_ was determined, and samples were analyzed by western blot using anti-VjbR or anti-RibH1 polyclonal antibodies. Results are representative from one of two independent experiments. (B) Analysis of *vjbR* promoter activity. Strain *B. abortus* P*_vjbR_*-*lacZ* was cultured in TSB and shifted to MM1 at pH 5.5 in the absence (gray circles) or presence (black circles) of 5 mM urocanic acid. Subsequently, β-galactosidase activities were determined at the indicated times. Values are means ± standard deviations of duplicate samples from a representative of two independent experiments.

β-galactosidase activity of strain *B. abortus ΔvjbR* P*_vjbR_*-*lacZ* (data not shown) or *B. abortus* P*_vjbR_*-*lacZ* (Fig. 2B) showed no significant variation under any of the tested media, which indicated that *vjbR* promoter activity is also constitutive under these different low-nutrient acidic conditions. Therefore, similarly to that observed in the stationary phase of growth in TSB, these results indicated that urocanic acid and pH 5.5 triggered VjbR protein expression by post-transcriptional regulatory mechanisms. Western blot analyses of bacteria incubated between 0 and 4 hours showed no expression of VjbR at pH 4.5 or 7.0, both in the absence or in the presence of urocanic acid (data not shown). On the other hand, at pH 5.5 the protein was detected in the presence of urocanic acid from 80 min onwards (Fig. 3A). β-galactosidase determinations showed no differences of *vjbR* promoter activity at pH 5.5 between treatments with or without urocanic acid over time (Fig. 3B), thus confirming that induction of VjbR protein expression is rapidly achieved independently of transcriptional activity.

### Other virulence-related proteins are also induced at pH 5.5 in the presence of urocanic acid

**Figure 4:**
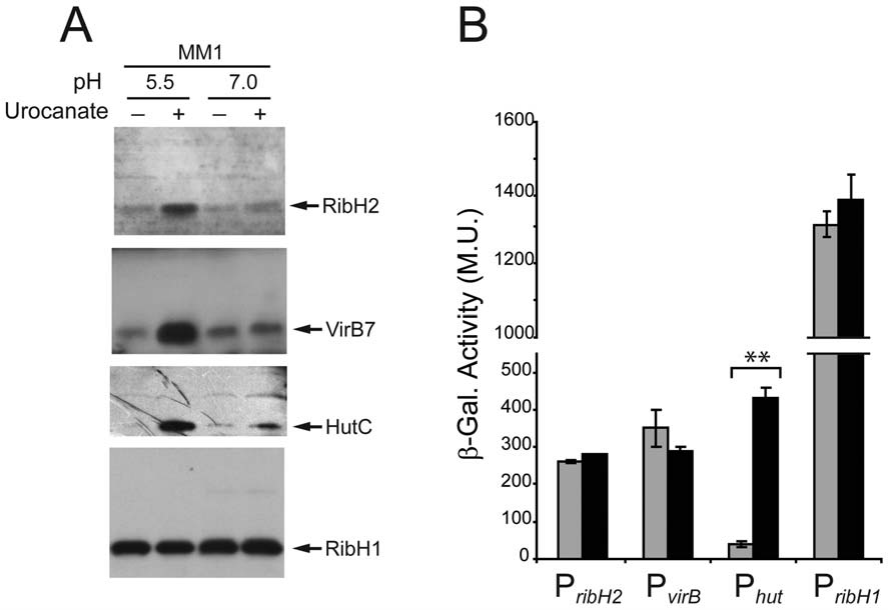
Urocanic acid also induced expression of other *Brucella* virulence-associated proteins. (A) Samples corresponding to *B. abortus* 2308 grown in TSB and shifted to MM1 at pH 5.5, or 7.0 in the absence or presence of 5 mM urocanic acid; were analyzed by western blot using anti-RibH2, anti-VirB7, anti-HutC or anti-RibH1 antibodies. (B) Analysis of *ribH2* (P*_ribH2_*), *virB* (P*_virB_*), *hut* (P*_hut_*), or *ribH1* (P*_ribH1_*) promoter activity. Strains *B. abortus* P*_ribH2_*-*lacZ*, *B. abortus* P*_virB_*-*lacZ*, *B. abortus* P*_hut_*-*lacZ*, or *B. abortus* P*_ribH1_*-*lacZ* were grown in TSB and shifted to MM1 at pH 5.5 in the absence (gray bars) or presence (black bars) of 5 mM urocanic acid. Subsequently, β-galactosidase activities were determined. Values are means ± standard deviations of duplicate samples from a representative of three independent experiments. **, *P*<0.01.

The above-mentioned observations led us to hypothesize that other proteins contributing to the intracellular survival of *Brucella* may also be induced in response to the same stimuli that trigger VjbR expression. To assess this possibility, extracts corresponding to bacteria incubated at pH 5.5 or 7.0, in the absence or presence of urocanic acid, were analyzed by western blot using antibodies against selected specific virulence-associated proteins. RibH2 is a second LS isoenzyme that, unlike RibH1, is required for intracellular replication of *Brucella* in macrophage cell lines [23]. As shown in Fig. 4A, RibH2 was induced under the same conditions that triggered expression of VjbR, whereas no effect was observed at pH 7.0, or when urocanic acid was absent. Moreover, western blot analyses revealed that VirB7, a component of the T4SS machinery, was also induced at pH 5.5 in the presence of urocanic acid but not in the other conditions tested (Fig. 4A). VirB7 protein expression was not achieved in the *vjbR* mutant background under these conditions (data not shown), indicating that the urocanic acid-mediated expression of VirB7 at pH 5.5 is VjbR-dependent, which is consistent with previous reports which showed that deletion of *vjbR* abrogates *virB* transcription and consequently prevents VirB protein expression [9], [14], [27].

**Figure 5:**
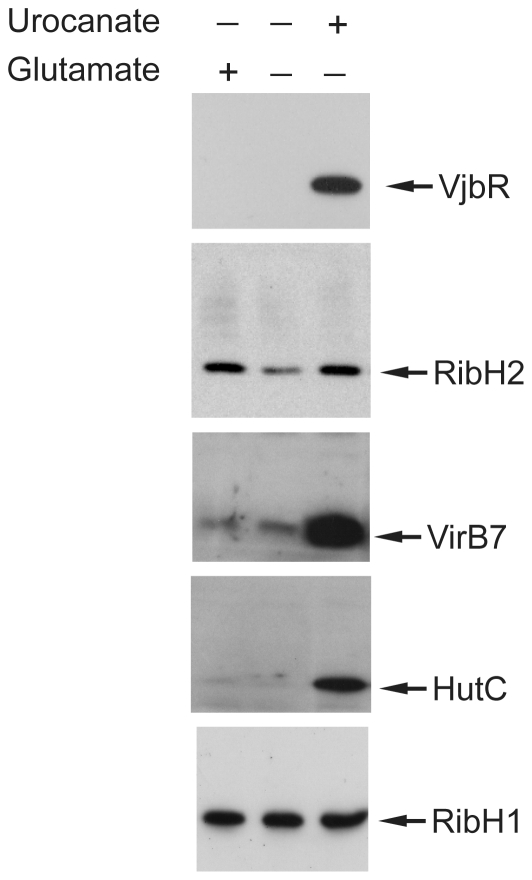
Comparison between glutamic acid and urocanic acid as inducer signals. Strain *B. abortus* 2308 grown in TSB and shifted to MM1 at pH 5.5, in the presence of 5 mM glutamic acid, 5 mM urocanic acid, or without the addition of any carbon source, as indicated. Subsequently, OD_600_ was determined, and samples were analyzed by western blot using anti-VjbR, anti-RibH2, anti-VirB7, anti-HutC or anti-RibH1 polyclonal antibodies.

Finally, we also analyzed the levels of HutC, a protein whose expression is known to be induced by urocanic acid at the transcriptional level, both in *Brucella* and in other systems [12], [30]. Fig. 4A shows that, although the strongest induction was observed at acidic pH, urocanic acid triggered expression of HutC at both pH values tested. These results confirmed the expected dependence of urocanic acid for induction of HutC, which was different from that of RibH2, VirB7, and VjbR, since induction of expression of these virulence-associated proteins not only required urocanic acid but also a specific pH value of 5.5. Specificity of the urocanic acid-mediated induction of protein expression was evaluated by assaying the addition of glutamate, a primary carbon source. As shown in Fig. 5, western blot analyses showed that glutamate was able to induce expression of RibH2 but not that of VjbR, VirB7 or HutC, indicating that the addition of urocanic acid is a specific requirement for all the assayed proteins except for RibH2.

**Figure 6:**
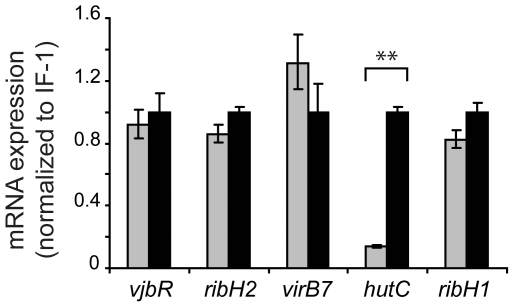
Quantitative real-time PCR analysis of the effect of urocanic acid on gene expression. Strain *B. abortus* 2308 was grown in TSB and shifted to MM1 at pH 5.5, in the absence (gray bars) or presence (black bars) of 5 mM urocanic acid. Subsequently, total RNA was isolated and processed for quantitative real-time PCR to analyze the levels of *vjbR*, *ribH2*, *virB7*, *hutC*, and *ribH1* transcripts. Values are expressed as relative mRNA fold differences between treatments with (black bars) or without (gray bars) 5 mM urocanic acid, indicating means ± standard deviations of duplicate samples from a representative of two independent experiments. **, *P*<0.01.

To evaluate whether the observed modulation of protein expression correlated with changes in transcriptional promoter activity, single-copy chromosomal transcriptional fusions between the *ribH2*, *virB*, *hut* or *ribH1* promoter and the *lacZ* reporter gene were constructed. The transcriptional fusion carrying strains were incubated at pH 5.5 in the absence or presence of urocanic acid, and β-galactosidase activities were determined. As expected, urocanic acid induced transcriptional activation of the *hut* promoter (Fig. 4B), which correlated with the increase of HutC protein levels (Fig. 4A). We also observed a correlation between transcriptional activity of the *ribH1* promoter (Fig. 4B) and the RibH1 protein levels (Fig. 4A), which remained unchanged in response to urocanic acid. In contrast, transcriptional activity of the *ribH2* and *virB* promoters was not affected by urocanic acid (Fig. 4B), whereas the protein levels of RibH2 and VirB7 changed in response to the inducer (Fig. 4A). These observations indicated that, similarly to that observed for VjbR, incubation at pH 5.5 in the presence of urocanic acid triggered expression of RibH2 and VirB7 by post-transcriptional regulatory mechanisms. In order to confirm these results by a different method, and to assess whether the post-transcriptional regulation of expression of VjbR, RibH2 and VirB7 was due to modulation of translational efficiency or by controlling mRNA stability, the relative levels of each transcript were determined by quantitative real-time PCR in bacteria incubated at pH 5.5 in the absence or presence of urocanic acid. As shown in Fig. 6, the *hutC* transcript levels were induced in response to urocanic acid, confirming that this compound induces transcriptional activation of the *hut* promoter. On the other hand, the *ribH1* mRNA levels were unaffected by urocanic acid (Fig. 6), consistently with the RibH1 protein determinations and the activity of its respective promoter (Fig. 4). These results indicated that both the HutC and RibH1 protein expression levels were a result of transcription and subsequent translation with no intermediate regulatory events. In contrast, the *vjbR*, *ribH2*, and *virB7* mRNA levels showed no variation in response to urocanic acid (Fig. 6), which demonstrated that the observed induction of VjbR, RibH2 and VirB7 protein expression was due to post-transcriptional regulatory mechanisms that do not involve modulation of mRNA decay. Taken together, these results demonstrated that expression of these three intracellular survival-related proteins is tightly regulated by mechanisms that do not involve activation of the transcriptional machinery, and act post-transcriptionially in response to the convergence of two different environmental cues related to the intracellular lifestyle of *Brucella*.

## Discussion

Besides acting as a central regulator of virulence due to its activity on *virB* expression, VjbR is involved in transcriptional regulation of many targets including flagellar components and outer membrane proteins [14], [31]. Accordingly, mutations affecting this LuxR-type protein lead to pleiotropic effects that alter several properties of the bacterial surface [31]. Furthermore, high-throughput analyses demonstrated that, either directly or indirectly, VjbR participates in the control of expression of hundreds of genes in bacteria cultured in rich medium [21], [28]. Thus, both number and type of functions affected by VjbR constitute substantial evidence for assigning a role for this QS-related element as a global regulator. Given that VjbR is able to bind to DNA and activate transcription in the absence of any autoinducer signal, one might speculate the existence of mechanisms that control the VjbR protein levels to induce VjbR-mediated transcription and also to prevent constitutive or inappropriate spatio-temporal expression of its target genes. To test this hypothesis, here we performed analyses of expression of the *vjbR* gene at different levels. Experiments carried out in rich medium showed that the *vjbR* promoter exhibits a constitutive transcriptional activity, whereas the VjbR protein was expressed only in the stationary phase of growth (Fig. 1), indicating that in *B. abortus* a post-transcriptional mechanism is involved in the regulation of this LuxR homolog. Our observation that *B. abortus ΔvjbR* exhibited a constitutive *vjbR* promoter activity higher than that of the wild type strain (Fig. 1B) differs from results reported in other works [9], [27], [28]. However, these previous analyses used experimental conditions not comparable to our study (i.e., multicopy plasmid constructs, heterologous systems or a *B. melitensis* background). Nevertheless, in view of the differences observed between *B. abortus* and *B. melitensis*, it can not be ruled out that regulation of the *vjbR* promoter probably differs among *Brucella* species.

Experiments performed in minimal media also showed a constitutive *vjbR* promoter activity under different acidic and metabolic conditions, whereas the VjbR protein expression was induced only with urocanic acid and pH 5.5 at 80 min of treatment. The same effect was observed at longer times, although, compared to the other conditions, the gradual loss of viability occurred between 0 and 4 h at pH 4.5 without urocanic acid may have differentially influenced protein expression. These findings demonstrated that expression of VjbR is tightly regulated, which supports a model whereby *Brucella* modulates the concentration of this LuxR homolog in the bacterial cell as a way to control transcription of the VjbR target genes in response to specific stimuli. The fact that VjbR is expressed only under a restricted set of conditions indicates that such regulatory mechanisms involve perception and integration of specific environmental signals of different nature.

It is well established that acidic pH is a critical physicochemical parameter affecting expression of the *virB* genes and intracellular survival of *Brucella* [3], [5]. Thus, it is not surprising that the virulence-associated regulator VjbR is also induced upon acidification, which may act as one of the signals that tell the bacterium that is inside the host cell. However, our experiments showed that VjbR protein expression is induced at pH 5.5 but not at pH 4.5 (Fig. 2), whereas the intravacuolar pH of BCVs reaches values of about 4.5 within the first 60 min of intracellular infection, and it is maintained by several hours at the same levels [3]. If we assume that this acidification process implies a decrease of pH from values close to physiological, *Brucella* necessarily has to deal with intermediate pH values within BCVs before achieving the steady state at pH 4.5. Thus, it can be speculated that during the first minutes after internalization, a transient exposure to pH 5.5 could induce a pulse of VjbR protein expression, which may serve as the stimulus to trigger downstream VjbR-mediated transcriptional activation. Nevertheless, it can not be ruled out that additional unknown parameters could account for the observed differences of optimal pH for VjbR expression in MM1 compared to the intracellular conditions reported by Porte *et al* [3]. It is also worth noting that the pH requirements for the post-transcriptional induction of VjbR, RibH2 and VirB7 protein expression (pH 5.5) (Fig. 2) differ from that previously reported for both the IHF- and HutC-mediated transcriptional induction of the *virB* genes (pH 7.0 and 4.5, respectively) [11], [12], which support the hypothesis that a sequential succession of intracellular pH-related events is necessary for *Brucella* to properly express its virulence-associated genes. Previous reports that compared protein expression at different pH values showed that the VirB proteins are induced under acidic conditions in *B. suis* [32], [33], whereas in *B. abortus* and *B. melitensis* such an acid-mediated induction was observed to a lesser extent [33]. Here, in the absence of urocanic acid, we did not observe an increase of VirB7 expression upon acidification. However, we assayed protein expression under nutrient limitation conditions using minimal media that lack carbon and nitrogen sources [11]. Thus, bacterial species, media composition, and the specific pH values we assayed are different from those used in other reports [9], [32], [33], and therefore our results are not strictly comparable to all of those previous analyses.

In addition to specific pH values, the second environmental variable required to trigger expression of VjbR under nutrient limitation conditions is the presence of urocanic acid (Fig. 2). It is worth mentioning that we assayed this compound because of the regulatory link existing between histidine catabolism and transcription of the *virB* genes, which was evidenced by molecular interactions between HutC and both the *virB* and *hut* promoters [12]. Consistently with early reports which showed that *Brucella* is able to incorporate and metabolize urocanic acid as efficiently as other primary carbon sources [34], [35], we have previously observed that *Brucella* is able to grow in minimal medium at pH 5.5 using urocanic acid but not histidine, its direct precursor [12]. The results presented here showed that, in addition to VjbR, the simultaneous exposure to pH 5.5 and urocanic acid also triggered the expression of VirB7 and RibH2 (Fig. 4), two proteins essential for the intracellular survival of *Brucella* [6], [23]. The addition of glutamate, a primary carbon source, was sufficient to induce the expression of RibH2, but not that of VjbR or VirB7 (Fig. 5); indicating that regulation of these proteins is not controlled by the same mechanisms. This latter observation suggests that regulation of expression of RibH2 is connected to signals related to the activity of central metabolism, regardless of the nature of the carbon source added. In contrast, induction of VjbR and VirB7 showed the specific requirement of urocanic acid, suggesting the existence of mechanisms that specifically detect incorporation and/or consumption of this compound. Taken together with previous observations [12], the results reported here demonstrate that urocanic acid can act as a signal for the induction of several virulence-associated genes, and reinforce the notion that this compound plays a central role in the connection between *Brucella* virulence and metabolism. In view of these observations, we cannot rule out the possibility that urocanic acid probably participates in the control of a wider regulatory network and, accordingly, it will be interesting to investigate whether other intracellular survival-related proteins of *Brucella* are also induced in response to this compound. Also, it will be important to determine whether an *in vivo* source of urocanic acid provides the signal to modulate virulence gene expression in *Brucella*, or whether the signaling pathway activated in cultured bacteria may be switched on *in vivo* by other structurally-related molecule, or by a particular metabolic state of the bacterium.

Altogether, our findings constitute evidence that may contribute to extend our understanding of the physiological responses of *Brucella* during the cross-talk with the host. In this work, the overall analysis of promoter activity, protein amounts, and mRNA levels demonstrated that urocanic acid and pH 5.5 induced expression of VjbR in a post-transcriptional manner (Fig. 2 and 4), leaving open the possibility that a regulatory mechanism acts by modulating the translational efficiency of the transcripts, or by controlling proteolysis of VjbR itself. This could represent a way for *Brucella* to elicit responses without involving recruitment or activation of the transcriptional machinery, allowing the bacterium to rapidly adapt and survive in the changing environment encountered within the host. Such responses involve integration of two different environmental stimuli (i.e., pH 5.5 and the presence of urocanic acid), which is a property that may confer robustness to these regulatory circuits, since no activation would be achieved in the absence of one of these two required signals.

## Supporting Information

Figure S1 **Effect of pH on bacterial viability in MM1.** (A) Wild-type strain *B. abortus* 2308 was grown in TSB until OD_600_ <1.5. Subsequently, cultures were centrifuged and bacteria were resuspended in medium MM1 at pH 4.5, 5.5, or 7.0; in the absence (open bars) or in the presence (gray bars) of 5 mM urocanic acid. After 4 h of incubation, OD_600_ was measured and CFU/ml was determined by serial dilution and plating on TSB agar for bacteria incubated at the indicated pH values. Values are means ± standard deviations of duplicate samples from a representative of two independent experiments. (B) Effect of pH 4.5 on bacterial viability at different times. Wild-type strain *B. abortus* 2308 was grown in TSB until OD_600_<1.5 and shifted to MM1 at pH 4.5 in the absence (open circles) or in the presence (gray circles) of 5 mM urocanic acid. At the indicated times, OD_600_ was measured and CFU/ml was determined by serial dilution and plating on TSB agar. Values are means ± standard deviations of duplicate samples from a representative of two independent experiments. **, *P*<0.01; *, *P*<0.05.
(TIF)
