# Supplementary figures and images for: Expression of VjbR under Nutrient Limitation Conditions Is Regulated at the Post-Transcriptional Level by Specific Acidic pH Values and Urocanic Acid
Source: PLoS One. 2012 Apr 17;7(4):e35394. doi: 10.1371/journal.pone.0035394 (PMC3328445; doi:10.1371/journal.pone.0035394)

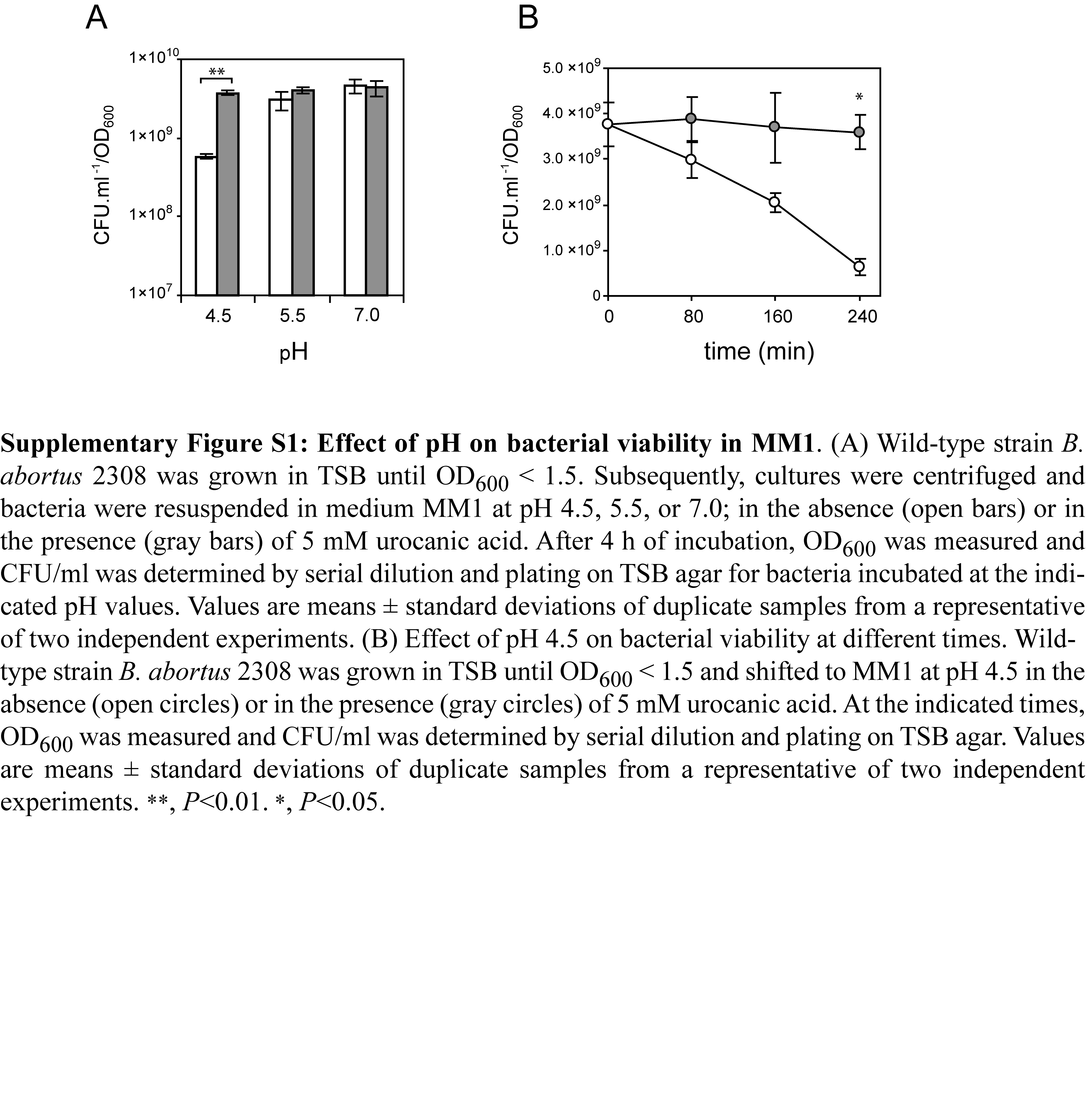

Supplement: Figure S1 — Effect of pH on bacterial viability in MM1. (A) Wild-type strain B. abortus 2308 was grown in TSB until OD600 <1.5. Subsequently, cultures were centrifuged and bacteria were resuspended in medium MM1 at pH 4.5, 5.5, or 7.0; in the absence (open bars) or in the presence (gray bars) of 5 mM urocanic acid. After 4 h of incubation, OD600 was measured and CFU/ml was determined by serial dilution and plating on TSB agar for bacteria incubated at the indicated pH values. Values are means ± standard deviations of duplicate samples from a representative of two independent experiments. (B) Effect of pH 4.5 on bacterial viability at different times. Wild-type strain B. abortus 2308 was grown in TSB until OD600<1.5 and shifted to MM1 at pH 4.5 in the absence (open circles) or in the presence (gray circles) of 5 mM urocanic acid. At the indicated times, OD600 was measured and CFU/ml was determined by serial dilution and plating on TSB agar. Values are means ± standard deviations of duplicate samples from a representative of two independent experiments. **, P<0.01; *, P<0.05. (TIF) [file pone.0035394.s001.tif]
